# A Comparison of Squatting Exercise on a Centrifuge and With Earth Gravity

**DOI:** 10.3389/fphys.2018.01759

**Published:** 2018-12-05

**Authors:** Timothy Piotrowski, Jörn Rittweger, Jochen Zange

**Affiliations:** Institute of Aerospace Medicine, Muscle and Bone Metabolism, German Aerospace Center, Cologne, Germany

**Keywords:** human centrifuge, space physiology, exercise physiology, resistance exercise, oxygen consumption

## Abstract

**Purpose:** Long-duration space missions require countermeasures against the muscular wasting and cardiovascular deconditioning associated with microgravity. Replacing gravitational acceleration by means of centrifugation is a promising alternative as it challenges all physiological systems at once. The aim of this study is to examine the metabolic energy costs of squatting on a centrifuge in comparison with squatting in an upright standing posture under natural gravity.

**Methods:** 24 subjects (11 male, 13 female) performed continuous squatting exercise for 9 min with increasing cadence (10, 12, and 15 squats min^-1^). This was done under three conditions: Upright under natural gravity and lying supine on a centrifuge at two radii (2.5 and 3.5 m) at 1 *g* of centrifugal acceleration at the subject’s average center of mass during the exercise.

**Results:** Generally, subjects did not suffer from motion sickness. Exercise under natural gravity led to a higher Δ V’O_2_/body mass (7.1 ± 2.0, ml min^-1^ kg^-1^, mean ± SD) compared with exercise on the centrifuge (6.1 ± 1.6, ml min^-1^ kg^-1^, mean ± SD). Exercise efficiency was also reduced under natural 1 *g* at 28.2 ± 1.0% compared to 40.4 ± 1.5% on the centrifuge. As expected, oxygen consumption increased with increasing cadences. The Coriolis effect had a negligible impact as there was no significant difference in V’O_2_ between the two radii. However, during centrifugation and upward movement the right leg was more loaded than the leg left and vice versa during downward movement (centrifuge running clockwise looking down, so to the subjects’ right).

**Conclusion:** The lower V’O_2_ on the centrifuge may be attributed to the unloading of trunk muscles while subjects were lying on the sled, which in the upright condition leaning against the sled were still working to stabilize the torso. Subjects tolerated high rotational rates combined with exercise very well.

## Introduction

Exploration will always remain one of humanity’s defining features and a driving force for technological advancement, and space continues to be an intriguing frontier. The micro-g environment leads to severe deconditioning (Sides et al., 2005) which, to date, can only partially be ameliorated. Thus, for sustained human health during long-term spaceflight, countermeasures have to be employed (Pavy-Le Traon et al., 2007).

One currently investigated strategy is the replacement of gravity by means of centrifugal forces caused by acceleration of the body mass in a rotating environment. In this context centrifugal forces are colloquially named “artificial gravity” (Clement et al., 2015). One implementation of such a countermeasure is a short arm centrifuge. With radii of about 5 m, even current launch vehicles could transport short arm centrifuges into low earth orbit. The principle behind this would be to mimic the natural mechanical environment as it is present on the earth’s surface as closely as possible and thereby prevent deleterious effects of microgravity (Iwase, 2005; Rittweger, 2007; Caiozzo et al., 2009).

It has been proposed that “static” centrifugal acceleration, i.e., without any movement within the rotating environment, could be beneficial in itself (Clement et al., 2016). In support of that claim, daily short arm human centrifugation (SAHC) conserved vertical jump performance in a 5-day bed rest study (Linnarsson et al., 2015; Rittweger et al., 2015). However, there were no benefits by SAHC in that study on biochemical markers of bone turn-over or nitrogen metabolism. Therefore, some exercise may be required within the SAHC, which has been called an “artificial gravity gym” (Vernikos, 1997; Moore et al., 2005). The efficiency of centrifugation on improving performance of musculature and the cardiovascular system was generally better when centrifugation was combined with resistance training (Yang et al., 2007) or cardiovascular training (Bonjour et al., 2010; Yang et al., 2010), respectively, in comparison with centrifugation or exercise alone. One of the greatest advantages of centrifugation is that it challenges all physiological systems at the same time. The negative effects of micro-gravity also manifest as space flight-associated neuro-ocular syndrome (SANS), a complex syndrome with potentially far reaching consequences which cannot be counteracted adequately with current countermeasures (Mader et al., 2011; Lee et al., 2018). A centrifuge could improve on this.

**FIGURE 1:**
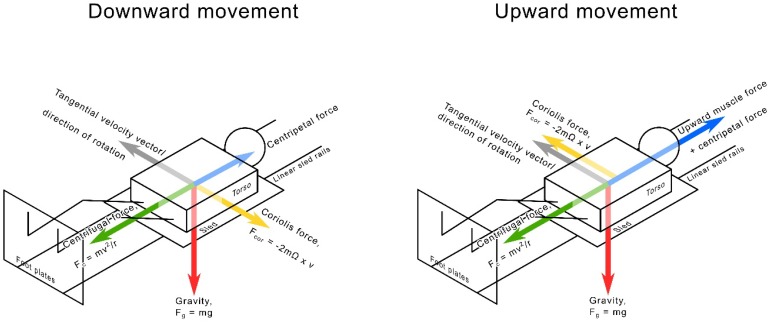
Free body diagram showing main forces acting on the body during upward and downward movement on the centrifuge. The Coriolis force (yellow) is shown alternating from the subject’s left to their right side. The length of the force arrows is not representative of their relative magnitude and only serves to illustrate the principle behind dynamic exercise on a centrifuge. F_c_ – centrifugal force, m – mass, v – velocity, r – radius, F_g_ – force due to gravity, g – acceleration due to gravity, F_cor_ – Coriolis force, Ω – angular velocity.

The unique environment on a centrifuge with regards to acceleration poses some interesting questions concerning movement and exercise in such a system. With acceleration increasing linearly with the radius, a subject lying supine with the feet facing outward on a centrifuge experiences less force on their upper body and arms than on their legs. Additionally, the Coriolis effect generates forces acting sideward on the mass of a body moving along the radius, altering circumferential speed (Figure 1). These extraneous forces could hinder exercise performance to some degree.

A recent study with a similar background examined the biomechanics of squatting on the centrifuge (Duda et al., 2012). Our study aims to measure the metabolic energy turn-over by means of respiratory oxygen uptake comparing squat exercise under natural gravity in a standing position with squats while lying supine in a short arm centrifuge. With the Coriolis effect acting on a large body part such as the head and torso, motion sickness was anticipated to be an important problem.

Hypotheses:

(1) Oxygen uptake (relative to body mass) is affected by the experimental g-condition, i.e., centrifugation at long or short radius or in the natural gravity condition.
(2) Oxygen uptake increases with increasing cadence of squatting.

## Materials and Methods

### Subjects

The 24 subjects were selected from the pool of applicants based on the inclusion/exclusion criteria (Table 1, biometric data can be seen in Table 2). After giving their written informed consent, all subjects underwent a comprehensive medical evaluation at the in-house physician’s office. These screening tests included medical history, blood analysis of standard clinical parameters and an orthostatic tolerance test (*Schellong I*, blood pressure and heart rate measurements while lying down and after standing up quickly).

**Table 1:** Inclusion/exclusion criteria.

Criterion	Acceptable range
Age, years	20–50
Mass, kg	50–100
Stature, m	1.55–1.90
BMI	18–25

*Other exclusion criteria: smoking in the past 6 months; hyper-/hypotension; propensity for syncope; propensity for motion sickness; relevant psychiatric diagnosis which could interfere with the study; relevant bone fractures; osteoporosis; (osteo-)arthritis, particularly of the knee, hip and spine; diabetes type I or II; pregnancy*.

**Table 2:** Subject biometric data.

Sex	Mean stature, m (SD)	Mean mass, kg (SD)	Mean age, years (SD)
Men (*n* = 13)	1.78 (0.07)	78.4 (8.6)	29 (6)
Women (*n* = 11)	1.68 (0.06)	61.2 (5.8)	27 (7)
Total (*n* = 24)	1.74 (0.09)	70.5 (11.3)	28 (7)

This study adheres to the Declaration of Helsinki in its seventh revision (2013) and was approved by the Ethics Committee of the Medical Council of North Rhine (Ethikkommission der Ärztekammer Nordrhein, Düsseldorf, Germany, application no. 2016027).

### Study Design

The study took place in the :envihab research complex at the German Aerospace Center (DLR) in Cologne.

As the purpose of this study was to compare exercise under natural gravity and on a centrifuge, special care was taken to design the experimental setup in such a way as to make the two gravitational environments as comparable as possible.

**FIGURE 2:**
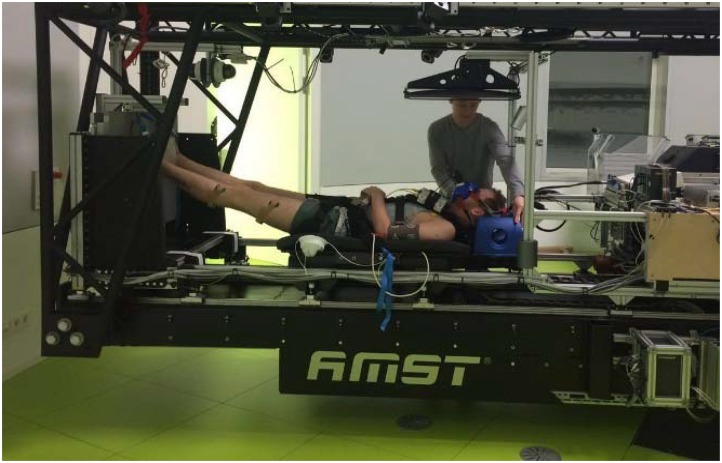
The sled system used on the centrifuge. The visual feedback system screen can be seen above the subject’s head. Written informed consent for the publication of this image was obtained from all persons present in the photograph.

**FIGURE 3:**
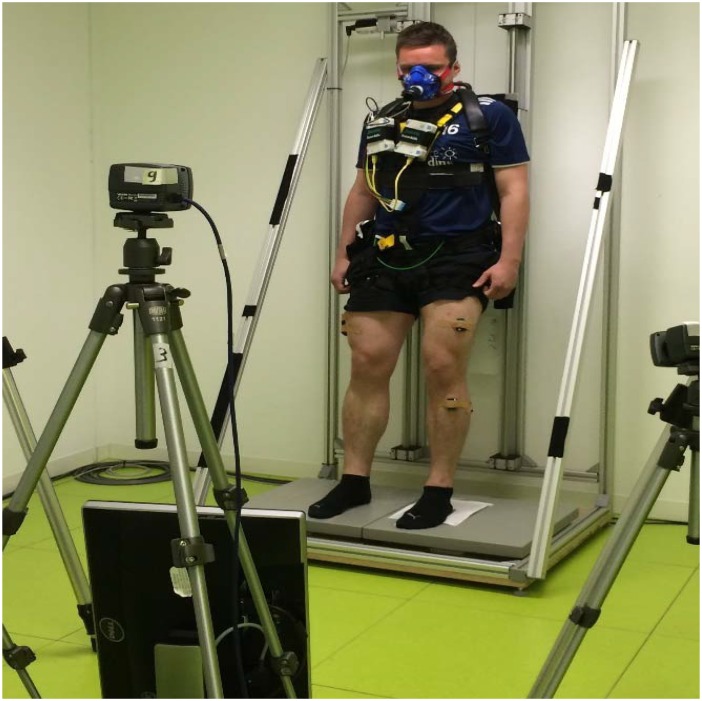
The sled system used under natural gravity. The yellow harness is not a safety feature, it only serves to equalize the mass on the natural gravity setup. Written informed consent for the publication of this image was obtained from all persons present in the photograph.

The centrifuge used was of the short-arm variety. The subjects laid supine with their head oriented to the axis of rotation and with the torso fixed to a sled which was able to move radially outward (Figures 2, 3). The feet were placed on a plate that was set at a radius of either 2.5 m or 3.5 m. Furthermore, the feet were suspended using slings at the outer rim of the centrifuge.

All subject performed three sessions of guided squat exercise in the following order of conditions:

(1) Natural gravity, standing and leaning against a sled
(2) Long radius centrifugation (3.5 m), lying supine
(3) Short radius centrifugation (2.5 m), lying supine

The order of conditions was assumed to represent an increasing risk of dropping out due to motion sickness. Therefore, this order was not permutated in between subjects to get at least some data on conditions 1 and 2 from every subject.

**FIGURE 4:**
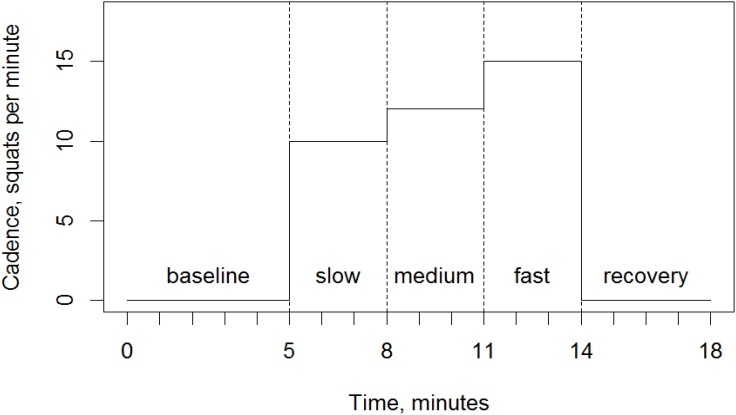
Exercise protocol for all g-conditions. Duration of baseline, squat exercise at three different cadences and the final period of recovery.

The protocol of each session started with a 5 min baseline measurement followed by a 9 min long period of squat exercise, which was subdivided into three 3 min long segments with increasing cadences of 10, 12, and 15 squats min^-1^, respectively. The protocol ended with a period of 4 min of recovery (see Figure 4).

At condition 1, subjects exercised with their body weight plus the weight of the equipment. On a centrifuge at a constant velocity of rotation the acceleration experienced by a mass increases with increasing distance to the axis of rotation. Therefore, during squats on a centrifuge, the ground reaction force increased when subjects moved their body toward the periphery by bending their knees.

The following procedure was performed to get the average exercise load on the centrifuge to be as close as possible to condition 1 (natural gravity, standing upright): At both radii, the subjects bent their knees until they reached the mid-point of a squat using the feedback system explained in detail later. Then the centrifuge was accelerated until the ground reaction force reached the value previously determined as total weight for the exercise under condition 1. For the long radius, the rotation velocity was on average 17 RPM, while at the short radius on average 22 RPM were used.

### Determination of Mechanical Power

During squatting ground reaction forces were recorded for each foot by a pair of force plates (AMTI, BP400600 (centrifuge)/OR6-6-2000 (natural gravity), Watertown, MA, United States). The motion of the body was recorded using a displacement sensor mounted to the sled (AK Industries, CD60, Lohmar, Germany). Analog signals of force and displacement were recorded at 1000 Hz and a precision of 1 N or 1 mm, respectively, using a Vicon Motion capture base station (Vicon Motion Systems, Inc., Centennial, CO, United States). During the last 2 min of each cadence period the current power was calculated as the product of force (N) and the motion velocity (m s^-1^). For the knee extensor musculature, the downward (eccentric) motion resulted in negative values of power and the upward (concentric) motion in positive values. Finally, for each cadence interval the absolute mean of eccentric and concentric power were calculated.

### Controlling Squatting Cadence and the Range of Motion

In this study, the cadence and the range of motion during squats had to be standardized across all subjects and all conditions. To this end, a visual feedback system was used. The subjects could see their current displacement as well as a reference signal on a screen in front of them. This allowed them to adjust their movement speed and range so that their squats were consistent on both the centrifuge and under natural gravity. The top position was defined as having the knees fully extended but not locked and the hips in contact with the sled while the bottom position had the subjects’ knees flexed at 90°. The feedback system software was calibrated for each subject, as body height influences the range of motion during a squat.

### Measurement of Respiratory Oxygen Consumption

A mobile spirometry device was used (BD CareFusion Oxycon Mobile, San Diego, CA, United States). It consisted of two modules fixed to the subject’s chest using a harness. The spirometer was calibrated at the start of each day with a manufacturer-supplied calibration gas of known composition as well as the local temperature and pressure. The spirometer used employed breath-by-breath capture and analysis. It measured V’O_2_, V’CO_2_ (both in ml min^-1^), ventilation rate and heart rate. The data were synchronized using manually triggered markers at the beginning and end of exercise as well as at each transition from one cadence to the next.

Values of V’O_2_ were averaged over the last 2 min of each cadence interval, when V’O_2_ approximately reached a steady state. The data from the 1st min of each interval were disregarded as the values fluctuated a lot before reaching a steady state.

The metabolic efficiency (%) of exercise was estimated based on an assumed caloric equivalent of 20.9 J ml^-1^ O_2_ and an assumption of the lower energy costs for eccentric contraction compared with concentric exercise. The assumed factor for the energy cost of eccentric exercise was derived as a compromise of a factor determined by ^31^P-magnetic resonance spectroscopy examinations on a single muscle group (Ryschon et al., 1997) and a factor determined by measuring V’O_2_ during downhill walking (Minetti et al., 2002). The metabolic efficiency (%) of exercise was finally calculated using the following model equation:

$$\text{Efficiency}(\%)=100\times \frac{\text{concentric power + 0.3 ecentric power}}{\Delta {\text{V}}^{\prime }{\text{O}}_{2}\frac{20.9}{60}}$$

### Electromyography (EMG)

A wireless EMG system was used (Delsys Trigno Lab Wireless EMG, Natick, MA, United States). Four sensors were placed only on the left leg above the *vastus lateralis, biceps* *femoris, gastrocnemius* *lateralis*, and *tibialis* *anterior* muscles. Data of all EMG channels were recorded at 1000 Hz on a Vicon Motion capture base station (Vicon Motion Systems Inc., Centennial, CO, United States). The application sites were shaved and cleaned with alcohol before the sensors were placed. The amplitude of the EMG signal was normalized to an EMG signal measured during isometric MVC. MVC measurements were performed at the beginning of each day. Using a dynamometer (IsoMed 2000, D&R Ferstl GmbH, Hernau, Germany) and vocal commands the subjects completed three repetitions of isometric knee extension and flexion as well as plantar flexion and dorsiflexion of the foot, with the maximal values being used for the MVC calculation.

During squats the EMG amplitudes were evaluated separately for the downward (eccentric) and the upward (concentric) movement.

### Total Hemoglobin and Muscular Oxygenation

A wireless device for NIRS (Artinis Medical Systems PortaMon, Elst, Netherlands) was placed above the *vastus lateralis* muscle on the right leg. Data were recorded at 10 Hz. During the recording the beginning and end of exercise and the changes of cadence were labeled by manually set markers. NIRS was used to determine leg muscle concentrations of O_2_Hb and HHb given in μmol L^-1^. tHb (μmol L^-1^) and the TSI (%) were calculated according Eqs 1 and 2.

(1) $$\text{tHb}={\text{O}}_{2}\text{Hb}+\text{HHb}$$

(2) $$\text{TSI}=100\times {\text{O}}_{2}\text{Hb}/\text{tHb}$$

The changes in tHb and TSI were calculated relative to the baseline measurement 1 min before exercise start. Just as in the V’O_2_ measurement, only the last 2 min of each of the three cadence intervals were used.

### Motion Sickness

After each centrifuge run the subjects were asked if any motion sickness was experienced using a simple verbal query. Only in the absence of motion sickness did we continued with further tests.

### Statistical Analysis

Statistical analysis we used the software SPSS (IBM SPSS Statistics Version 21).

Using linear mixed effect (LME) models, we tested the fixed effects cadence (10, 12, and 15 repetitions per minute), experimental g-condition (AG at short radius, AG at long radius, natural gravity) and gender (male, female). Furthermore within the LME-function the effects of g-conditions were tested pairwise, applying *t*-tests and a correction of denominator degrees of freedom. For all tested variables it turned out that the effects in the two AG conditions were not significantly different. Therefore the statistical tests were repeated using the simplified factor g-condition (AG, natural gravity). Results of LME are given as *F*(numerator degrees of freedom/degrees of freedom) *f*-value, *p*-value The results of subsequent *t*-test are given as (corrected denominator degrees of freedom), *p*-value. A value of *p* < 0.05 was accepted for significance.

## Results

**FIGURE 5:**
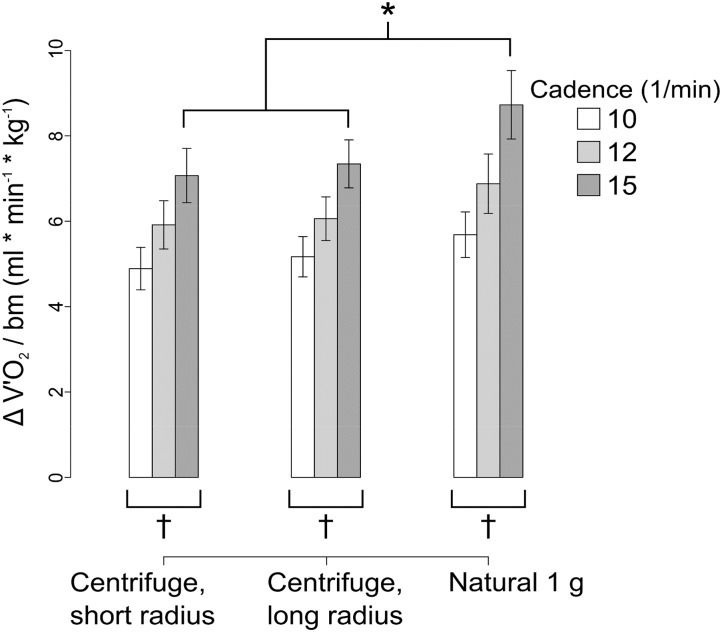
Additional oxygen consumption over baseline normalized to body weight showing mean ± SE. ^∗^*p* < 0.05 for g-condition. ^†^*p* < 0.05 for cadence. Higher oxygen consumption under natural gravity can be seen.

Under all three g-conditions V’O_2_ normalized to body weight increased with higher cadences (LME: *F*(2/122.073) 54.522, *p* < 0.001, Figure 5). V’O_2_ values on the centrifuge at the two radii did not differ significantly from one another (*t*-test: (132.776), *p* = 0.262). Under natural gravity V’O_2_ was, on average, 14% higher than the summarized V’O_2_ measured under both centrifuge conditions (LME: *F*(1/105.081) 18.124, *p* < 0.001). The absence of a significant difference between the two radii implies a negligible impact of the Coriolis effect, as it is stronger at the smaller radius.

**FIGURE 6:**
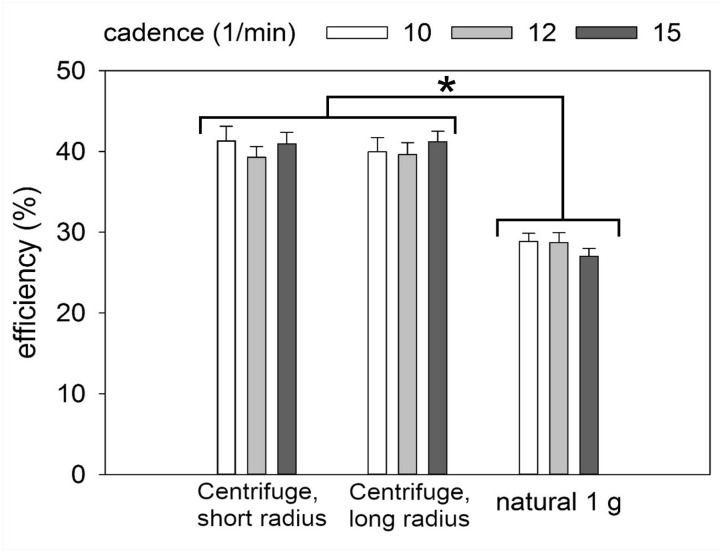
Metabolic efficiency showing mean ± SE. ^∗^*p* < 0.05. Efficiency was significantly higher on the centrifuge compared to natural gravity.

On average, across all cadences, efficiency was 12% lower under natural 1 *g* than on the centrifuge (LME: *F*(2/120.950) 149.881, *p <* 0.001, Figure 6). The two centrifuge radii did not prove to be significantly different from an efficiency perspective. Cadence was also not a significant factor.

**FIGURE 7:**
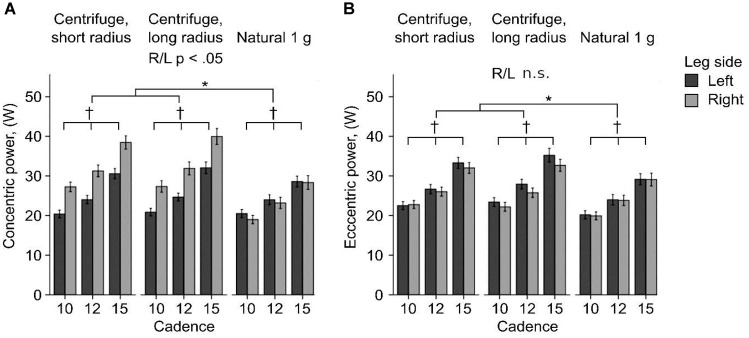
Concentric **(A)** and eccentric **(B)** power of the right and left leg showing mean ± SE. ^∗^*p* < 0.05 for g-condition. ^†^*p* < 0.05 for cadence. Generally more power was produced on the centrifuge, here differences between the left and right leg are also present, but only during concentric movement.

On the centrifuge both legs produced more power than under natural gravity (difference in mean about 8 W, LME: *F*(2/135.911) 28.276, *p <* 0.001, Figure 7). Additionally, differences between the left and right leg were only observed during centrifugation. During downward (eccentric) movement under rotation to the subject’s right, the left leg is loaded more than the right leg and produces approximately 1 W more, on average. During upward (concentric) movement the right leg is loaded more, producing approximately 5 W more than the left leg. While the difference between right and left leg forces is significant during concentric movement on the centrifuge, during eccentric movement only a trend can be seen and there is no significant difference between right and left, just as under natural gravity. Cadence had a significant effect on power in both movement types, with higher cadences linked to higher power (LME: *F*(2/119.999) 73.977, *p* < 0.001).

**FIGURE 8:**
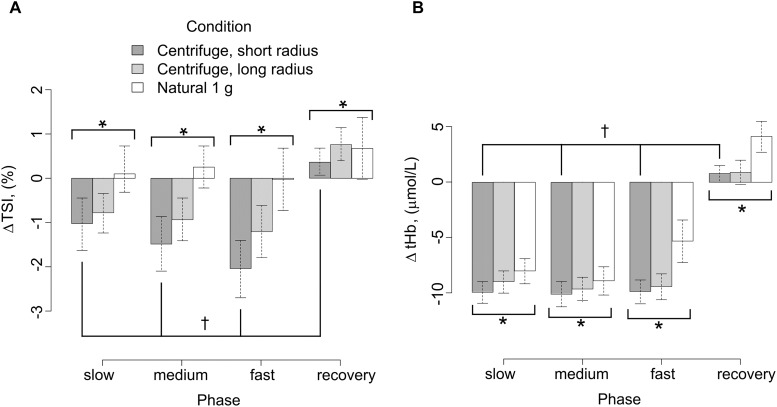
**(A)** Near infrared spectroscopy results Δ TSI of the right *vastus lateralis* between the baseline 1 min before exercise compared to during exercise, showing mean ± SE. ^∗^*p* < 0.05 for g-condition. ^†^*p* < 0.05 for cadence. **(B)** Near infrared spectroscopy results Δ tHb of the right *vastus lateralis* between the baseline 1 min before exercise compared to during exercise, showing mean ± SE. ^∗^*p* < 0.05 for g-condition. ^†^*p* < 0.05 for cadence.

At baseline, which was measure 1 min before the start of the exercise (see Figure 4), in the right *vastus lateralis* muscle the relative hemoglobin oxygenation given as TSI (mean ± SD) was a little higher on the centrifuge (65.5 ± 3.1%, long radius; 67.0 ± 5.2%, short radius) than under natural gravity (63.3 ± 4.7%, Figure 8A). However, only the baseline values found under natural gravity and on the short radius condition were significantly different from each other (*t*-test: (32.804), *p* < 0.05). During exercise only very small changes in TSI occurred. During exercise under both conditions of centrifugation ΔTSI showed different effects than under natural gravity (LME: *F*(1/57,330) 11.474, *p* < 0.01) On the centrifuge, ΔTSI decreased by up to 2% as the protocol progressed before returning back up to normal levels during the recovery period. Under centrifugation, this was up to almost 1% more on the short radius than on the long one. Under natural gravity, average ΔTSI was not altered by squatting at any of the three cadences and during recovery from exercise there was only a slight, non-significant increase.

During the baseline measurement, centrifugation did not result in significantly different levels of tHb (μmol L^-1^ tissue) in comparison with natural gravity (65.5 ± 3.1 long radius, 67.0 ± 5.3 short radius, 63.3 ± 4.7 natural gravity, Figure 8B). During the initial muscle contractions of exercise under all g-conditions ΔtHb dropped by values between 4 and 10 μmol L^-1^, with a significantly larger change in ΔtHb on the centrifuge in general compared with the natural g-condition (LME: *F*(1/62.127) 4.367, *p* < 0.05).

Electromyography amplitudes normalized to MVC were only measured on the left leg, which during concentric contraction on the centrifuge produced less power than the right leg due to the Coriolis effect (see also Figure 7A). In all muscles and in both directions of movement, cadence and gender didn’t affect the EMG amplitudes.

**FIGURE 9:**
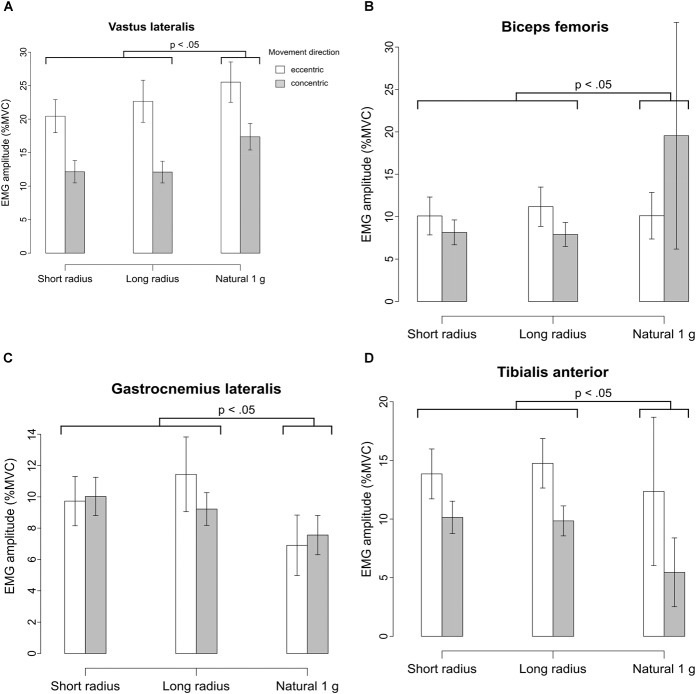
**(A)** Normalized EMG signals of the left *vastus lateralis* as a percentage of MVC amplitude during eccentric/concentric contraction under the three g-conditions. Showing mean ± SE. This muscle showed particularly high activation during eccentric movement under all conditions. **(B)** Normalized EMG signals of the left *biceps femoris* as a percentage of MVC amplitude during eccentric/concentric contraction under the three g-conditions. Showing mean ± SE. Highest activation was measured during concentric movement under natural gravity. **(C)** Normalized EMG signals of the left *gastrocnemius lateralis* as a percentage of MVC amplitude during eccentric/concentric contraction under the three g-conditions. Showing mean ± SE. The muscles of the lower leg were activated more strongly on the centrifuge. **(D)** Normalized EMG signals of the left *tibialis anterior* as a percentage of MVC amplitude during eccentric/concentric contraction under the three g-conditions. Showing mean ± SE.

During concentric contraction under natural gravity (Figure 9A), the normalized EMG amplitude of the *vastus lateralis* was about 5% higher than under both AG conditions on average (LME: *F*(1/59.047) 39.375, *p* < 0.01) During eccentric contraction, the difference in EMG amplitude between natural gravity and AG was only 3% (LME: *F*(1/120.810) 6.598, *p* < 0.05). EMG amplitudes measured on the short and long radius did not differ significantly. Activation during concentric contraction was lower than during eccentric contraction. Gender was not a significant factor.

The *biceps femoris* (Figure 9B) muscle showed about 12% higher activation during concentric contraction under natural gravity than on average on the centrifuge (LME: *P*(1/62.994) 13.342, *p* < 0.01). During eccentric contraction the g-condition did not significantly affect the EMG-amplitude of the *biceps femoris* muscle.

The muscles of the lower leg, *gastrocnemius lateralis* and *tibialis anterior* showed a higher activation on the centrifuge of approximately 2% for both muscles’ overall activation, with the *tibialis anterior* showing higher activation during eccentric contraction (Figures 9C,D).

## Discussion

Our hypothesis concerning oxygen consumption was confirmed, during squatting exercise on the centrifuge metabolic energy turn-over measured as V’O_2_ differed from squatting under natural gravity and was lower. Nevertheless, this was slightly surprising as we thought the added Coriolis force was likely to lead to a higher V’O_2_. Although the centrifugal force was adjusted to body weight in the halfway squatting position, the measured mechanical power was slightly higher on the centrifuge than under natural gravity, especially during upward (concentric) movement. Since the higher metabolic energy turn-over during squatting under natural gravity is linked with an additional slightly lower mechanical output of the legs, upright squatting exercise under natural gravity increases body energy costs for other purposes than the mere mechanical work of lifting.

One principal difference between the two experimental setups which could explain the lower V’O_2_ on the centrifuge in comparison with upright standing was a different need to stabilize the trunk. On the centrifuge, the torso was lying supine. It was supported by the moving sled platform and fixed in place by a safety harness. During upright squatting under earth gravity the torso was also held and guided by the sled platform with a safety harness. However, in the upright posture the body was still carrying and stabilizing itself, which may explain the higher metabolic turn-over in the upright posture. Even though we could not establish a sizable effect of the Coriolis force on oxygen consumption in this study, we cannot exclude such effects under a different configuration concerning radius or rotational rate.

During squatting on the centrifuge the Coriolis effect resulted in a sideward force on the subjects’ bodies. As the body moved up, it was deflected in the direction of rotation, in this case to the subject’s right, thereby shifting more weight onto the right leg. Although the head, the trunk and the feet were prevented from moving sideways by appropriate fixation, subjects reacted to the sensation of the sideward forces by developing mechanical power in their leg musculature.

Unfortunately, EMGs were only recorded on the left leg, as the right leg was used for NIRS recording. Under natural gravity, both the left *vastus lateralis* muscle and the left *biceps femoris* muscle showed a higher EMG amplitude than on the centrifuge.

However, on the centrifuge we measured higher EMG amplitudes of the musculature of the lower leg, i.e., the *gastrocnemius lateralis* muscle and *tibialis anterior* muscle. They aided in stabilizing the body from side to side to counteract the Coriolis effect by means of stiffening the ankle joint by co-activation. As the knee cannot rotate in the sagittal axis, this rotation induced by the Coriolis force has to take place in the ankle and hip joints.

They may also be activated more because of the gravity gradient. At the lowest point in the squat the acceleration due to rotation was highest and the Coriolis effect was lowest because the subject was almost not moving at all radially. Thus, a high activation of both the right and left calf muscles was needed. While calf muscle activation was higher on the centrifuge, thigh muscle activation was lower and as the thighs are more massive this is congruent with the overall lower oxygen consumption.

This study involved only a short overall time on the centrifuge of about half an hour total per subject. Longer periods of training and adaptation to centrifugation and Coriolis forces could lead to more even loading of the right and left leg as the subject learns to anticipate the shifting of their body. Alternatively, the direction of rotation could be switched back and forth between training sessions in order to maintain equal loading on both legs.

The left *vastus lateralis*, which was measured by the EMG sensor, was activated less during upward movement on the centrifuge because the right *vastus lateralis* was likely used and activated more because of the shifting of body weight due to the Coriolis effect. The *biceps femoris* was activated strongly under natural gravity in order to stabilize the knee and hip joints together with the co-activation of the *vastus lateralis.* Even though they are antagonists, both muscles are required for movements such as rising from a squatting position.

In order to further examine differential leg loading and activation on the centrifuge due to Coriolis forces, future studies should incorporate EMG sensors on both legs. Our study’s statements on muscle activation are limited because only one side was measured and, as can be seen from the results of the foot reaction forces, differential loading takes place on the centrifuge.

The tHb measurement from the NIRS sensors shows an effect of the gravity gradient. The short radius had the largest drop in tHb and natural gravity the lowest. The shorter the radius (and the taller the subject), the higher the difference in acceleration between head and toes (Δ*g*), in some cases this difference was larger than 1 *g*. This gradient forced blood into the lower extremities before the exercise as the subject was lying in a supine position, but with the centrifuge already rotating. When the exercise started, more blood was present in the muscles, so more blood could be displaced by the muscle pump leading to a greater drop in tHb where the gravity gradient was highest, that is on the short radius.

All results concerning tissue saturation showed only very small changes, suggesting equilibrium of oxygen supply and demand. The TSI showed the largest drop during exercise on the short radius, no change under natural gravity and the long radius values in-between these two. This trend is consistent with the gravity gradient. The gravity gradient is highest on the short radius, slightly lower on the long radius and non-existent under natural gravity. A lower TSI can be due to more consumption or less flow into the muscle. As this was the right leg and we saw higher power on the right leg during centrifugation, a higher consumption seems likely. On the other hand, a similar mechanism as seen in the tHb could be present, with a higher drop due to more preloading with O_2_Hb due to the gravity gradient. The same limitations concerning one-sided measurements apply to the NIRS measurements as to the EMG measurements.

As a limitation of our study design the fixed order of the experimental conditions may have caused some adaptation effects to physiological responses to exercise. As mentioned before the order of experiments was chosen by the increasing risk of motion sickness to increase the number of finished test runs, in case the subjects would poorly tolerate squatting on the centrifuge. Moreover, some but not all of the subjects were familiar with the stress of passively lying on a centrifuge from other studies. However, for all subjects this was the first time that they were performing squatting exercise on the centrifuge. The tests were all done on the same day, which could have led to increasing fatigue. However, the time between runs was approximately 15–30 min due to setup and calibration, which would have reduced this effect if present.

Exercise on the centrifuge proved much easier to implement than expected by the research team. Even though rotational rates of up to 22 RPM (short radius) were used and the head of the subject was not stationary during the movement, no subject experienced kinetosis, with only one male subject complaining of slight discomfort. However, he was still able to complete the protocol after the lights were dimmed. This is consistent with previous studies which also showed only minor problems relating to motion sickness (Duda et al., 2012). During squatting subjects focused their vision on the screen used as a feedback system for motion control. This visual focus likely contributed to the high tolerance against motion sickness on the centrifuge.

We showed that metabolic energy turn-over during squatting at three different cadences was lower when subjects performed horizontal supine exercise on a short-arm centrifuge in comparison with upright squatting under natural gravity. Although the Coriolis effect had side effects on the eccentric and concentric external power, we could show that it did not have a significant impact on metabolic energy turn-over. This is a new aspect in centrifuge-based resistance training.

The fact that our concern related to motion sickness proved overestimated, especially considering the intrusive spirometry mask, makes us very hopeful for future studies testing the effectiveness of exercise on a centrifuge.

## Ethics Statement

Informed consent was obtained from all individual participants included in the study.

All procedures performed in studies involving human participants were in accordance with the ethical standards of the institutional and/or national research committee and with the 1964 Helsinki declaration and its later amendments or comparable ethical standards.

## Author Contributions

TP, JR, and JZ conceived and designed research. TP and JZ conducted the experiments. TP, JR, and JZ analyzed data. TP wrote the manuscript. All authors read and approved the manuscript.

## Conflict of Interest Statement

The authors declare that the research was conducted in the absence of any commercial or financial relationships that could be construed as a potential conflict of interest.

## Abbreviations

EMG: electromyography
HHb: deoxygenated hemoglobin
MVC: maximum voluntary contraction
NIRS: near infrared spectroscopy
O_2_Hb: oxygenated hemoglobin
RPM: revolutions per minute
SAHC: short arm human centrifuge
SD: standard deviation
tHb: total hemoglobin
TSI: tissue oxygenation/saturation index
V’O_2_: rate of oxygen uptake
